# A Case of Long-Tract Ileocolic Intussusception Secondary to Well-Differentiated Cecal Adenocarcinoma

**DOI:** 10.7759/cureus.52208

**Published:** 2024-01-13

**Authors:** Mohamad Ballan, Mahroo Aghababaei, An Guo Michael Chin, Dmitriy Kim

**Affiliations:** 1 Surgery, William Carey University College of Osteopathic Medicine, Hattiesburg, USA; 2 Surgery, St. John's Episcopal Hospital, Queens, USA

**Keywords:** adult intestinal invagination, ileocolic type, adult intussusception, cecal adenocarcinoma, ileocolic intussusception

## Abstract

Intussusception denotes the intricate phenomenon wherein one segment of the bowel undergoes invagination or telescoping into its contiguous distal segment. The ensuing invaginated segment may be propelled forward through peristaltic movements, potentially precipitating bowel obstruction or ischemia, culminating in necrosis of the affected bowel segment. Although the precise etiology of intussusception remains elusive, particularly in cases devoid of an identifiable lead point, dysrhythmic contractions and lymphoid hyperplasia have been implicated in the pathophysiology of this condition.

We present the case of an 86-year-old African American female with a past medical history of hypertension and asthma who presented to our emergency room with a seven-day history of worsening abdominal. The pain was described as sharp and intermittent, and it would worsen with every meal or drink. A physical exam demonstrated the right lower quadrant with vague abdominal tenderness, especially below the umbilical region. Computed tomography of the abdomen and pelvis revealed a long segment of ileocolic obstructing intussusception in the ascending colon, with a 2.6 cm solid mass serving as a lead point. Swift intervention ensued with an urgent exploratory laparotomy, culminating in a right hemicolectomy to excise the intussuscepted segment of the bowel. The pathological examination identified a well-differentiated adenocarcinoma of the cecum, categorized as T1N0M0, with all 20 resected lymph nodes yielding negative results.

This illustrative case presents a unique insight into a patient with ileocolic obstructing intussusception, caused by a well-differentiated adenocarcinoma acting as the lead point, a relatively uncommon occurrence in adults. Diagnosing intussusception in adults is challenging due to its nonspecific symptoms, which are similar to those of various other gastrointestinal disorders. Therefore, it is crucial for medical providers to be acutely aware of the possibility that adenocarcinoma can trigger obstructing intussusception in various parts of the bowel.

## Introduction

Intussusception is the telescoping of one bowel segment into its adjacent distal segment. The invaginated segment can then be propelled forward via peristalsis and potentially cause bowel obstruction or ischemia, leading to necrosis of the bowel segment [1,2]. The exact cause of intussusception is not well understood, but it has been linked to abnormal muscle contractions and lymphoid hyperplasia [1-3]. Adult intussusception makes up only five percent of all intussusception cases and is responsible for approximately 1% to 5% of bowel obstructions in adults [4,5].

Intussusception in adults presents with a variety of vague, non-specific symptoms such as abdominal pain, nausea, and vomiting, making the preoperative diagnosis challenging. Moreover, the lead points in adult cases are often attributed to carcinomas, polyps, benign neoplasms, diverticulosis, and other pathologic conditions. These are typically found intraoperatively and are responsible for over 90% of intussusception cases in adults [6-9]. It is important to note that roughly 57% of reported adult intussusception cases are related to a malignant tumor [10]. Although abdominal computed tomography (CT) scans are valuable in identifying intussusception, they have limited utility in distinguishing between malignant, benign, and idiopathic lead points [11]. The clear distinction in etiology and the challenges of making the diagnosis explain why intussusception necessitates a surgical intervention in the adult population. We report an interesting case of ileocolic obstructing intussusception secondary to a well-differentiated adenocarcinoma functioning as the lead point in an 86-year-old African American female.

## Case presentation

An 86-year-old African American woman with a history of hypertension and asthma presented to the emergency department with a seven-day history of worsening abdominal pain. The pain was described as sharp and intermittent, and it worsened with the consumption of any food or fluid. The patient also reported anorexia, nausea, and vomiting, including an episode of vomiting undigested food. The patient denied experiencing symptoms such as constipation, diarrhea, bloody or bilious vomiting, bloody or mucoid stools, or melena. The patient also denied any known history of colon cancer or other malignancies in her family. Furthermore, the patient had not undergone any routine gastrointestinal screenings, including colonoscopy or esophagogastroduodenoscopy (EGD), prior to this presentation.

**Figure 1:**
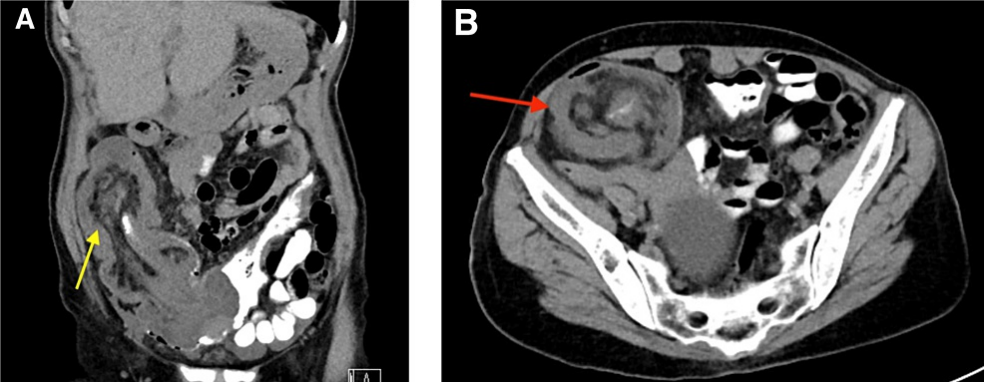
(A) Coronal view of the abdominal CT scan demonstrating ileocolic intussusception in the RLQ (yellow arrow). (B) Axial section showing the apple core appearance typical of Intussusception (red arrow).

On physical examination, the patient was found to have abdominal tenderness in the right lower quadrant. A rectal examination revealed soft, non-bloody stool in the rectum, but no palpable masses or visible blood were detected. Vital signs at the time of admission were as follows: blood pressure of 145/70 mmHg, pulse rate of 100 bpm, temperature of 97.9°F, and oxygen saturation of 100% while breathing room air. Laboratory results obtained on admission showed a hemoglobin of 10.9 g/dL, white blood cell count of 5,700/mm^3^, and platelet count of 334,000/mm^3^. A CT scan of the abdomen and pelvis revealed a long segment of ileocolic intussusception in the ascending colon, which appeared to be related to a 2.6 cm solid mass serving as a lead point (Figures 1A, 1B). As a result, the patient was placed on NPO status (Nulla per os; or “nothing by mouth”), rehydrated with IV fluids, and received IV pantoprazole sodium, acetaminophen, and morphine to control the pain. The patient was then admitted undergoing an urgent exploratory laparotomy and right hemicolectomy secondary to intussusception.

**Figure 2:**
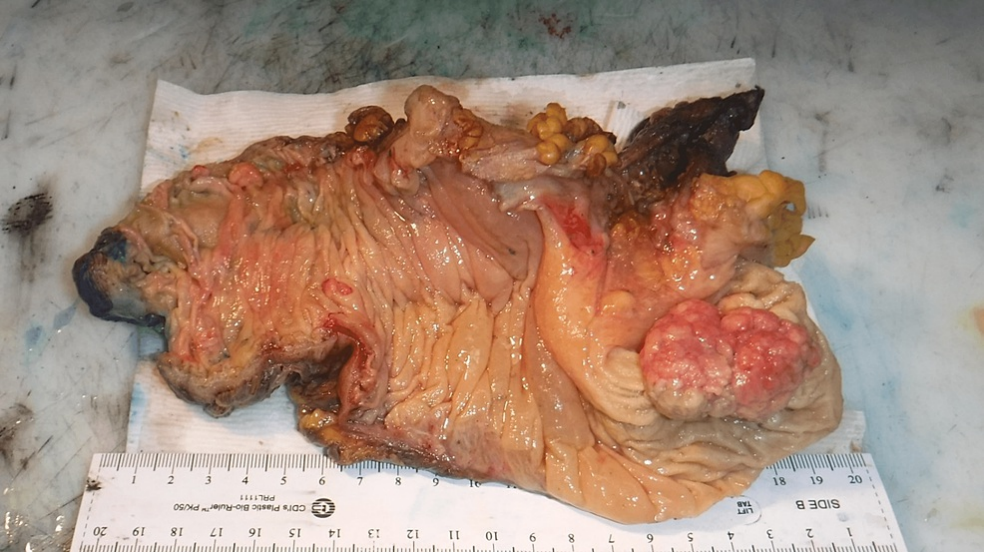
Macroscopic view of excised specimen showing ileocolic intussusception secondary to a growing mass in the cecum.

The patient underwent a midline laparotomy incision in the operating room. Upon exploration, a long 10 cm segment of ileocolic intussusception was identified (Figure 2). Due to the high suspicion of malignancy, no attempts were made to manually reduce the intussusception. The mesenteric lymph nodes were found to be enlarged at the ileocolic vessel pedicle, which was dissected and sent to pathology for microscopic examination. The peritoneum and the remainder of the abdomen were meticulously inspected, showing no evident metastatic masses. The omentum appeared thin but otherwise unremarkable. A right hemicolectomy was performed due to malignancy concerns. The small bowel was transected 10 cm proximal to the ileocecal valve to ensure an adequate margin from the malignancy site.

**Figure 3:**
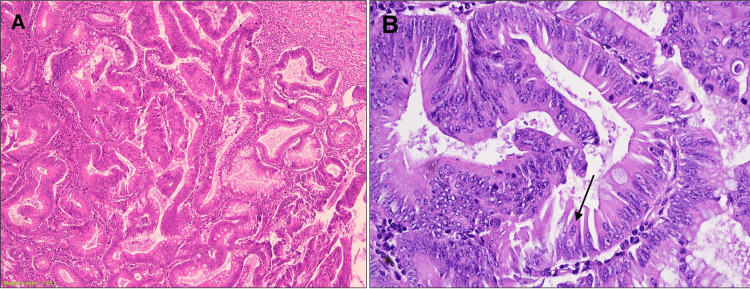
Representative microscopic photographs of well-differentiated adenocarcinoma (A) show a low-power view of a well-differentiated adenocarcinoma with no invasion of the serosa (H&E: x10). (B) High-power view (x40) of tumor cells displaying hyperchromatic chromatin, crowded nuclei and mitotic figures (black arrow).

Final grading and staging of the lead point was determined to be a well-differentiated adenocarcinoma of the cecum, T1N0M0, with no involvement of lymph nodes (0/20 negative) and no evidence of perineural or lymphovascular invasion (Figures 3A, 3B). The resection margins were clear. On gross examination, the specimen contained a pink-gray, firm polypoid tumor mass measuring 4.5 x 3.0 x 1.0 cm in the cecum (Figure 2). The tumor mass was found to be focally infiltrated into the submucosa but not through the serosa. The tumor was located 1.5 cm from the ileocecal valve, 8.5 cm from the proximal resection margin, and 8.0 cm from the distal resection margin. Additionally, three polyps were found in the right colon. The rest of the colon mucosa was moderate to markedly edematous. During the follow-up visits, the patient reported to be in good health and had fully recovered. Abdominal and chest CT and colonoscopy screenings were all negative for any signs of metastasis.

## Discussion

Anterograde intussusception occurs when a proximal part of the bowel slides into a distal part, leading to mechanical obstruction and/or bowel ischemia [3,6]. Retrograde intussusception, which is less common, can also occur in certain cases such as post-Roux-en-Y gastric bypass surgery. In this form of intussusception, the distal part of the intestine invaginates into the proximal part. The exact cause of retrograde intussusception is not fully understood, but there are reports that suggest post-op adhesions and/or staple lines serving as the iatrogenic lead points [12].

Based on its location, intussusception can be divided into four types: enteric, colonic, ileocolic, and ileo-cecal. When intussusception is confined to the small intestine, it is termed enteric (50% of adult cases). Intussusception that solely involves the large intestine is called colonic (32% of adult cases). Ileocolic and ileo-cecal intussusception accounts for about 18% of all adult cases. Ileocolic intussusception is characterized by the protrusion of the ileum into the ascending colon through the ileo-cecal valve. In ileocecal intussusception, the ileocecal valve serves as the lead point [6,12,13]. In this case report, we presented a rare glimpse of an ileocolic obstructing intussusception where a part of the terminal ileum telescoped into the cecum and ascending colon secondary to a cecal adenocarcinoma.

Adult intestinal intussusception, while infrequent, poses diagnostic and therapeutic challenges due to its manifestation through nonspecific signs and symptoms. A proactive measure to enhance preventive care involves patients undergoing routine colonoscopy screening and establishing ongoing care with primary care physicians. The utilization of a CT scan of the abdomen emerges as a pivotal and effective method for diagnosing intussusception, elucidating the presence or absence of a lead point [11,14]. Notably, laparoscopy has gained recognition in recent years as a valuable diagnostic and therapeutic tool in the nuanced management of both pediatric and adult intussusception [15-18].

Although surgical intervention remains the cornerstone of treatment for colonic intussusceptions in adults, certain reports advocate for manual reduction, albeit with considerable variation in patient selection criteria. This approach is particularly endorsed in post-traumatic and idiopathic cases devoid of a discernible pathological lead point, aiming to mitigate the risk of short bowel syndrome development [19,20]. While the preservation of bowel integrity is paramount, it is essential to acknowledge the substantial risks associated with this technique, including a heightened recurrence rate and the potential oversight of malignant mass lead points that may elude naked eye detection. Given the predominant association of adult intussusception cases with malignancy, a prevailing inclination favors the en-bloc resection of the intussusception without prior attempts at reduction or manipulation [19,20]. The preference for an en-bloc surgical approach is underscored by the concern that malignant cells may disseminate and seed other organs following the reduction of the intussuscepted segment. Similarly, in instances where bowel obstruction is evident, posing the risk of ischemia and necrosis, surgical resection has emerged as the treatment of choice [20].

## Conclusions

We present a unique case delineating ileocolic obstructing intussuception, an uncommon manifestation attributed to a well-differentiated adenocarcioma serving as the lead point in an 86-year-old African American female. The importance of early diagnosis and expeditious surgical intervention cannot be overstated, as it constitutes a pivotal measure in averting severe complications (bowel necrosis and sepsis) and augmenting patient survival in the context of malignancy-associated obstructive intussusceptions. Surgeons, in particular, assume a pivotal role in the comprehensive management of this condition and must exhibit proficiency in both the diagnosis and therapeutic strategies pertinent to adult intussusception, thereby ensuring optimal outcomes for their patients.
